# Targetless Calibration of Wide-Baseline and Wide-Angle Surround-View Fisheye Cameras Using Cylindrical Projection Model †

**DOI:** 10.3390/s26123622

**Published:** 2026-06-06

**Authors:** Gee Hoon Lee, Soon-Yong Park

**Affiliations:** School of Electronic and Electrical Engineering, Kyungpook National University, Daegu 41566, Republic of Korea; wlgns4123@knu.ac.kr

**Keywords:** wide-baseline fisheye camera, extrinsic calibration, cylindrical coordinate system, monocular depth estimation, multi-camera systems, autonomous driving

## Abstract

We propose a novel targetless extrinsic calibration method for wide-baseline and wide-angle fisheye cameras, which are mounted on a driving vehicle for surround view monitoring. Sequences of image frames from three fisheye cameras are obtained, and the object instance and depth around the vehicle are used for calibration. Thus, the proposed method can be applied to online vehicle camera calibration. Fisheye images are first transformed into the cylindrical coordinate system by considering the panoramic formation of the cameras. Then, the state-of-the-art object detection and monocular depth estimation models are applied to the cylindrical images. Vehicle instances matched across different views are reconstructed into 3D point clouds, and their depths are scaled by employing the pose geometry of the front camera. The per-point depths and global scale are then jointly optimized to achieve accurate cross-view alignment and extrinsic calibration. Experiments on both real-world and synthetic video datasets show that the proposed method achieves higher accuracy than COLMAP and DUSt3R under challenging conditions such as wide baselines and low frame rates, without requiring an artificial calibration target.

## 1. Introduction

Autonomous driving techniques continue to advance as research and development in both industry and academia. In such techniques, reliable perception of the surrounding environment is essential, which is typically achieved using various types of sensors such as cameras, LiDAR, and radar. Among them, fisheye cameras are widely integrated into surround-view perception systems because their 180° field of view enables near-complete 360° coverage with a relatively small number of sensors. To operate these multiple cameras as a unified perception system, the inter-camera extrinsic parameters must be accurately estimated. However, the severe geometric distortion inherent in fisheye images makes it difficult to directly apply conventional vision algorithms developed for pinhole cameras. In particular, depth estimation and inter-camera extrinsic calibration remain challenging, despite the practical advantages offered by fisheye cameras in achieving efficient coverage around the vehicle.

Due to these challenges, numerous studies have focused on improving the robustness and accuracy of the extrinsic calibration between fisheye cameras mounted on a vehicle. One of the representative methods is using chessboards or artificial patterns, which is supported by toolkits [1,2] such as Kalibr, OpenCV, and MATLAB. These toolkits rely on projection models [3,4,5,6] that account for the nonlinear distortion characteristics of fisheye lenses. Although this approach is widely used, the severe distortion of fisheye images often degrades corner detection accuracy, which in turn limits the reliability of the estimated parameters. Moreover, it requires a carefully controlled environment and is therefore typically restricted to the initial installation stage of cameras in the vehicle.

In practice, camera positions vary not only across different vehicle models but also within the same model due to installation tolerances, meaning that extrinsic parameters must be re-estimated for each case. This process imposes significant time and labor costs in mass production. Furthermore, the calibrated parameters can quickly become invalid in real-world driving due to vibrations, temperature changes, or collisions, making chessboard-based calibration methods impractical for long-term deployment in vehicles. Therefore, to overcome the limitations of chessboard-based calibration, several studies, such as [7,8], have proposed targetless autocalibration methods that operate directly in driving environments. However, these approaches typically require either accurate initial extrinsic parameters or auxiliary information such as vehicle odometry.

In contrast, this paper introduces a targetless automatic calibration method for wide-baseline and wide-angle fisheye cameras for vehicle surround view monitoring. The proposed method relies solely on camera observations without the need for any additional sensors or prior calibration. To solve the wide-baseline and wide-angle calibration problem, the proposed method leverages object detection and monocular depth estimation in cylindrical image space. Fisheye images are first projected onto a cylindrical image plane using the Enhanced Unified Camera Model (EUCM) [6]. This transformation makes the input images more compatible with perception networks, including YOLOv8 [9] for object detection and SPIdepth [10] for monocular depth estimation, which are typically applied to conventional perspective images.

Since there is only a 2D similarity transformation relationship in far distance object areas between the pinhole and cylindrical image spaces, we use the two deep networks without retraining. In addition, almost all pinhole-based models have been trained using datasets that already include 2D similarity images in each class to cover the object-centric rotation. Using YOLOv8, the surrounding vehicles are detected, and their feature points are matched across multiple camera views. To solve the distance ambiguity of the feature points between the different views, scale alignment is achieved by exploiting the ground geometry. Finally, geo-rectified 3D point clouds from multiple cameras are reconstructed and aligned to estimate the extrinsic parameters. This paper extends our previous work [11] by introducing optimization methods for more accurate scale alignment between different views and additional experiments for more comprehensive evaluation.

## 2. Related Works

In this section, we review the core techniques utilized in this work, along with related research on extrinsic parameter estimation for cameras.

### 2.1. Fisheye Camera Models

Fisheye cameras provide a wide field of view but exhibit significant distortion, which has motivated extensive research into accurate projection models. The equidistance model [3], which assumes that the distance from the image center to each pixel is proportional to the angle of the incoming ray, is one of the most basic projection models. Its simple formulation and computational efficiency are advantages, but it lacks the flexibility to model complex real-world lens distortions.

The Kannala–Brandt model, which is proposed in the same work [3], improves the equidistance model by expressing the radial distance as a high-order polynomial function of the incidence angle, allowing more accurate modeling of complex distortions. However, using higher-order polynomials requires numerical approximation for inverse projection and increases computational cost.

The Double Sphere (DS) model [4] projects 3D points onto two virtual spheres in sequence and maps them to the image plane. This approach models distortion with relatively simple equations while maintaining high accuracy, but its performance degrades under extreme distortion.

The Unified Camera Model (UCM) [5] maps image points by projecting onto a unit sphere followed by a central projection. While this allows multiple types of lenses to be represented with a single parameter, it exhibits low accuracy when applied to heavily distorted fisheye lenses.

The Enhanced Unified Camera Model (EUCM) [6] extends the UCM by introducing an additional parameter that controls the shape of the projection surface, thereby improving its capacity to represent various lens types with greater accuracy and flexibility. In this work, we adopt the EUCM to model the 3D-to-2D projection of the fisheye lens and construct a cylindrical projection model accordingly.

### 2.2. Object Detection Models

The inherent extreme distortion in fisheye images often deforms object appearances, presenting significant challenges for detection. Numerous studies have been conducted to address this problem. Some approaches aim to detect objects directly from fisheye images without image transformation. Griffiths and Dansereau [12] proposed RectConv, a distortion-aware convolutional filter, which improves detection performance without requiring image rectification. Similarly, Rashed et al. [13] introduced a fisheye-aware coordinate representation to enhance object detection accuracy.

Another approach transforms fisheye images into cylindrical projections before applying detection models. Plaut et al. [14] demonstrated that applying a conventional pinhole-based 3D object detection model to cylindrical images—without additional training—can achieve effective results without performance degradation.

Inspired by this, we apply a pinhole-based object detection model to cylindrical images without further adaptation. Specifically, we chose YOLOv8 [9], a state-of-the-art object detection model that provides a good balance between accuracy and computational efficiency and is widely used for real-time applications in various environments.

This choice is justified by a comparative study conducted by Gochoo et al. [15], who compared the performance of YOLO-based models on fisheye images without architectural modifications and showed that YOLOv8 achieved the best performance. This result suggests that YOLOv8 is robust to spatial distortions.

### 2.3. Monodepth Models

In this section, we review previous works on monocular depth estimation using fisheye images collected in road environments. Some studies have performed depth estimation directly on fisheye images without transformation. Lee et al. [16] improved estimation performance by introducing a Slanted Cylindrical Bin structure that reflects the geometric layout of the road. Zhao et al. [17] propose a self-supervised fisheye depth estimation framework that incorporates real-scale pose information to reduce scale ambiguity and improve accuracy.

These previous works typically adapt the model structure to account for fisheye distortion and train the model accordingly. In contrast, our method avoids retraining by first transforming fisheye images into a cylindrical coordinate system, then applying a powerful pre-trained pinhole-based monodepth model. However, applying the pinhole-based monodepth model to the cylindrical image causes severe geometric distortion in the 3D point cloud reconstruction. To solve this problem, we propose to re-project the monodepth image to the cylindrical space, which results in metric 3D reconstruction of the scene.

The model used in this paper is SPIdepth [10], which is trained in a self-supervised manner on KITTI Eigen split [18,19] and optionally fine-tuned on Cityscapes [20], and has achieved state-of-the-art performance across multiple benchmarks. In particular, SPIdepth demonstrates strong geometric consistency and high depth estimation accuracy in road scenes, making it well-suited for the objectives of this study. Furthermore, the zero-shot evaluation on the Make3D [21] dataset highlights SPIdepth’s remarkable generalization ability to unseen domains without retraining, suggesting that it can also perform robustly under the cylindrical projection employed in our framework.

### 2.4. Camera Pose Estimation

Camera pose estimation between multiple cameras can be categorized into methods that use a chessboard and those that do not. Chessboard-based calibration methods are supported by various toolkits, including Kalibr, OpenCV, and MATLAB. These approaches offer the advantage of producing relatively accurate results, but they require specific calibration targets such as chessboards and cannot compensate for shifts in camera positions that may occur during driving. Therefore, this study focuses on a targetless auto-calibration method that does not require a chessboard, in order to address such limitations.

Lee et al. [7] proposed a method for extrinsic calibration using coarse-to-fine random search and photometric loss optimization in the BEV (bird’s-eye view) domain, and reported high calibration accuracy in their experiments. However, their method relies on an initial estimate, and in their experiments, the initial values were intentionally set close to the ground truth (GT) to verify recoverability. Their experiments were conducted in the CARLA simulation environment [22], using relatively simple scenes without dynamic objects such as vehicles. Moreover, in cases where the common field of view between cameras lacked texture, a chessboard was added, revealing limitations in terms of generalization.

Heng et al. [8] proposed CamOdoCal, a framework that estimates extrinsic parameters between various types of cameras, including fisheye cameras, based on the vehicle odometry. This method performs high-precision calibration through iterative optimization and is highly practical as it can operate on real driving data without requiring calibration targets. However, it requires thousands of image frames to run optimization and bundle adjustment between the odometry from multiple cameras by using vocabulary trees of image features. For this pose optimization between cameras, the vehicle should move around so that there are feature overlaps between cameras. For this reason, this method needs more than one hour of computation time.

Wang et al. [23] introduced DUSt3R, a transformer-based dense feature matching network. This model demonstrates strong matching performance across various scenes and can be used for targetless camera pose estimation by predicting the relative pose between image pairs. Nevertheless, as it is based on the pinhole camera model, it struggles when the baseline and rotation between cameras are large, since the overlapping field of view becomes limited. Schönberger et al. [24] proposed COLMAP, a feature-based structure-from-motion (SfM) pipeline. COLMAP estimates camera extrinsic parameters using SIFT-based image matching and geometric verification, and supports a variety of camera models, including fisheye lenses. However, due to the nature of SfM, it can fail to register or recover frames in cases with large viewpoint changes or textureless scenes. To overcome these issues, we propose a method for estimating extrinsic parameters between fisheye cameras that can operate robustly even in challenging conditions, such as large temporal gaps between frames, wide baselines between cameras, and significant inter-camera rotations.

## 3. Proposed Method

### 3.1. Overview

**Figure 1:**
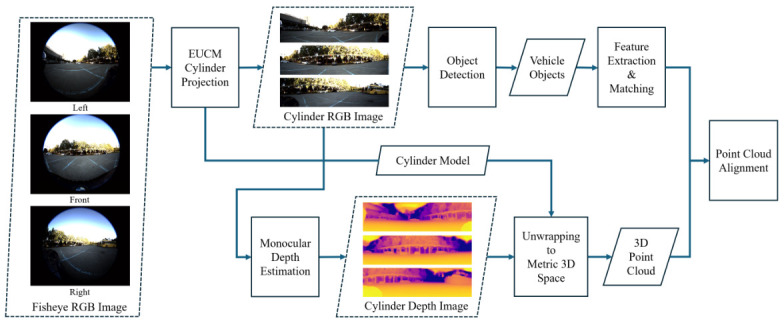
A flow diagram of the proposed method.

**Figure 2:**
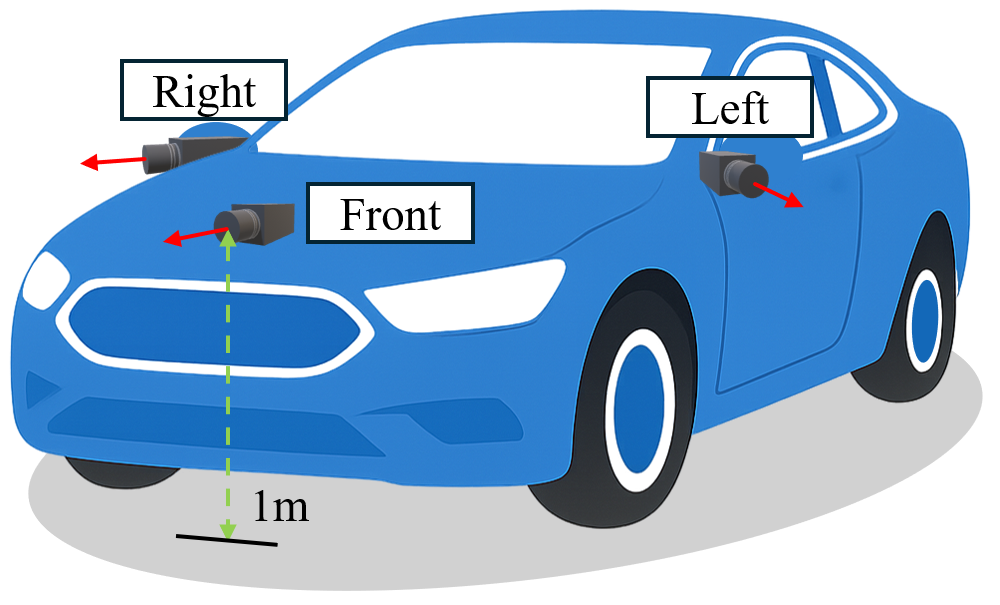
Illustration of fisheye camera installation on a vehicle.

This study proposes an automatic method of estimating relative external poses between multiple fisheye cameras. The proposed architecture is shown in Figure 1. Fisheye images are obtained from three cameras mounted on a moving vehicle. In the proposed formulation, we assume ideal inter-camera time synchronization, and images indexed by the same timestamp are treated as simultaneous observations. As shown in Figure 2, three cameras are mounted on the front, right, and left sides of the vehicle. The front camera is installed such that its *x*–*z* plane is assumed to be parallel to the ground, while the right and left cameras are rotated outward to achieve approximately 50–60% overlap with the front camera.

Among various fisheye camera models [3,4,5,6], we adopt the EUCM [6] model to project fisheye images into a cylindrical coordinate system. Cylindrical projection preserves the wide field of view of fisheye cameras and the 2D similarity relationship in far-distance objects with pinhole cameras. These similarities allow direct application of models trained on conventional pinhole images to the cylindrical projection images. Based on this transformation, YOLOv8 [9] is applied for surrounding vehicle detection, and SPIdepth [10] is used for monocular depth estimation without retraining or explicit distortion correction.

Detected vehicles from three cameras are used as matching targets across camera views. To incorporate the planar surface constraint of the road, each bounding box of a detected vehicle is extended downward by a factor of two along the image’s vertical axis. This expanded region includes both the vehicle and a portion of the road surface below it. This allows feature points to be sampled from both the vehicle body and the adjacent ground area. Feature points extracted from the upper and lower parts of the expanded regions are regarded as vehicle and ground feature points, respectively.

Matching information between the extracted feature points is used to identify the same vehicle across the front–right and front–left image pairs. Depth values of these points are estimated using the monocular depth model and are converted into 3D coordinates according to the cylindrical projection geometry. Assuming that the *x*–*z* plane of the front camera is parallel to the ground plane, ground feature points in the front view are used to compute a scale factor from the geometric relationship between the camera and the ground. This assumption can be generalized if the angle of the camera’s *x*–*z* plane with respect to the ground is known. The linear regression coefficient obtained from this process is uniformly applied to all matched feature points in the front, right, and left images, thereby achieving consistent scale alignment across all views. Using the scaled depth values, a 3D point cloud is generated for each camera, and the resulting point clouds are aligned to estimate the extrinsic parameters between cameras.

The key contributions of this paper are as follows:

- Introduce a targetless extrinsic calibration pipeline of wide-baseline and wide-angle fisheye cameras for surround view monitoring.
- Propose a 3D reconstruction method that interprets the estimated monocular depth values within the cylindrical coordinate system to generate distortion-corrected 3D point clouds.
- Achieve metric-scale correction of the 3D point clouds by leveraging the geometric assumption of the cameras.
- Estimates the relative pose between camera pairs through robust alignment of the reconstructed 3D point clouds.

### 3.2. Projection from Fisheye Image to Cylindrical Images

**Figure 3:**
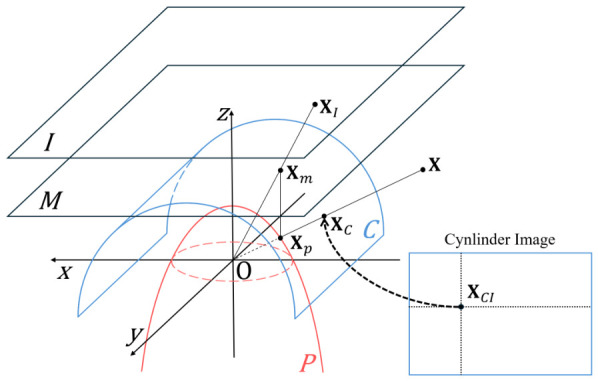
EUCM cylindrical projection model.

In this paper, we use the Enhanced Unified Camera Model (EUCM) to correct the distortion in fisheye images. We project a fisheye image to a cylindrical coordinate image using the projection relationship defined by EUCM. The geometric structure of this projection is illustrated in Figure 3. $X_{p}$ denotes the point obtained by projecting X onto the EUCM projection surface *P*. $X_{m}$ denotes the orthogonal projection of $X_{p}$ onto the z=1 normalization plane *M*. X represents a 3D point in the camera coordinate system. These notations follow the definitions introduced in the EUCM model [6]. *I* denotes the fisheye image.

An image point $X_{CI}$ in the cylindrical image is mapped to a 3D point $X_{C}$ on the 3D cylindrical surface *C* as follows.

(1a) $$\begin{array}{c} X_{C} \end{array}=\left(\begin{array}{c} x_{c}\\ y_{c}\\ z_{c} \end{array}\right),\;X_{CI}=\left(\begin{array}{c} u\\ v \end{array}\right),$$

(1b) $$\begin{array}{cc} x & =u-\frac{I_{w}}{2},\;y=v-\frac{I_{h}}{2},\;\theta =\frac{\pi \cdot x}{I_{w}} \end{array}$$

(1c) $$\begin{array}{cc} x_{c} & =r\cdot \sin\theta ,\;y_{c}=y,\;z_{c}=r\cdot \cos\theta \end{array}$$

In Equation (1), *x* and *y* mean the image-plane coordinates centered at the principal point. The angle θ is defined in the camera coordinate system, where negative *x* direction corresponds to 0° and the positive *x* direction to 180°. The cylindrical image is generated with the same resolution as the original fisheye image, and the radius value *r* used in the mapping equation is set to one-third of the image height to simplify computation. Each point $X_{CI}$ in the cylindrical image, mapped into 3D space as $X_{C}$, is then projected back to the point onto the fisheye image as $X_{I}$ using EUCM. Using this mapping relationship, pixel values are sampled from the original fisheye image to generate the cylindrical image. Instead of using the entire cylindrical image, only the central third is used, obtained by dividing the image vertically into three equally spaced areas. This selection removes regions such as the ground area close to the vehicle and the sky, which provide limited information for estimating extrinsic parameters, thereby reducing unnecessary computation.

### 3.3. Object Detection and Feature Matching

We estimate the extrinsic parameters between each camera pair by registering 3D point clouds reconstructed from matching feature points between the images. To extract these points, we use a combination of an object detection model and SIFT features [25]. As vehicles are the most frequently appearing objects in road environments, we first detect them using an object detection model, YOLOv8.

After vehicle detection, we expand the size of each bounding box by a factor of two in the positive *y*-direction to include a larger contextual region around the vehicle. SIFT features are then extracted within these expanded bounding boxes, and feature matching is performed between two images in a camera pair. For each pair of candidate vehicle boxes, we compute the mean descriptor distance over the matched features within the paired regions; if this average is below a threshold value $T_{m}=300$, the two boxes are considered to contain the same vehicle. From these confirmed pairs, we retain only the correspondences with individual descriptor distances no greater than another threshold $T_{r}=140$. Additionally, it is required that the relative positions of the matched features within their respective bounding boxes differ by less than ±10% between the two views.

**Figure 4:**
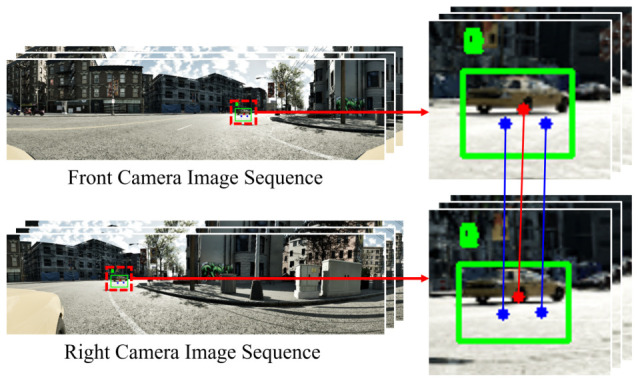
Feature matching example between front and right camera images.

The matched feature points are then divided according to their vertical positions (*y* values) within the bounding box: points in the lower half are classified as ground feature points, while those in the upper half are classified as vehicle feature points. An example of the resulting feature matching is shown in Figure 4, where red points indicate vehicle features from the upper half of the bounding box and blue points represent ground features from the lower half; the connecting lines visualize the correspondence pairs. To prevent redundant sampling across frames, newly detected feature points are constrained to maintain a minimum distance of 10 pixels from all previously collected points in the image. This matching and collection process is repeated over a fixed number of frames, after which the accumulated feature points from each camera are reconstructed into 3D feature points.

### 3.4. Monocular Depth Estimation and 3D Point Cloud Generation

In addition to using matching features between image pairs for extrinsic calibration, 3D structure in each camera scene is also employed. To obtain a 3D structure, we reconstruct 3D point clouds using a monocular depth estimation model combined with post-processing. Specifically, we apply the SPIdepth model to the images from which the features are extracted to obtain the depth values of each feature point. SPIdepth, trained on the KITTI Eigen split dataset [18,19], has been shown to generalize well to new domains, as demonstrated by its zero-shot performance on the Make3D dataset [21].

**Figure 5:**
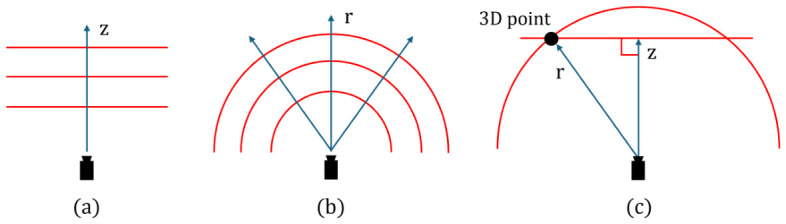
Interpretation of monocular depth estimation. (**a**) *z*, orthogonal distance from the image plane; (**b**) *r*, radial distance; (**c**) Difference between *r* and *z*.

SPIdepth is directly applied to cylindrical images, and we further apply post-processing to adapt the depth values to cylindrical coordinates, because the model is originally trained on pinhole images. In the pinhole model, as shown in Figure 5, depth *z* represents the distance in the direction perpendicular to the image plane. However, in cylindrical coordinates, the image plane corresponds to a curved surface in the shape of a semicylinder. Accordingly, instead of interpreting the depth as a distance along the camera’s *z*-axis, we apply a reinterpretation in which the depth is considered as the radius *r* of an expanding concentric circle centered at the camera, as shown in the center of Figure 5. This corresponds to a geometric model in which each column of the cylindrical image has its own local *z*-axis extending orthogonally from the curved image surface. The relationship between *r* and *z* is illustrated in Figure 5. Using the reinterpreted radius *r*, each feature point is then reconstructed into a 3D point according to (2).

(2) $$x=-r\cos\;\left(\frac{\pi u}{w}\right),\;y=r\left(\frac{v-\frac{h}{2}}{h}\right),\;z=r\sin\;\left(\frac{\pi u}{w}\right)$$

**Figure 6:**
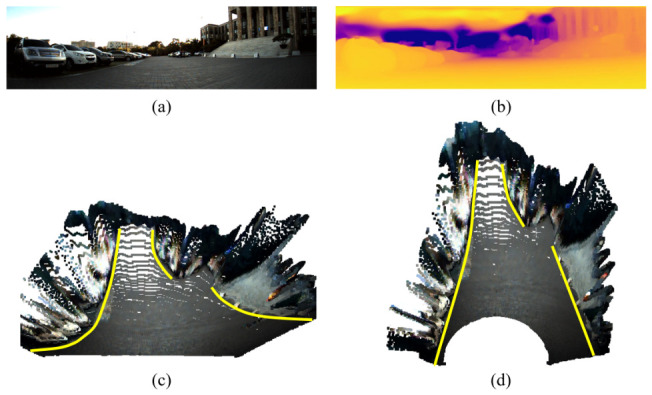
(**a**) RGB image; (**b**) Depth image; Comparison of 3D point cloud reconstruction with different depth interpretation, *z* (**c**) and *r* (**d**).

Equation (2) defines *r* as the estimated depth value, while *u* and *v* denote the pixel coordinates in the image space, and *w* and *h* indicate the image width and height, respectively. An example of the resulting 3D point cloud generated using this method is shown in Figure 6. In Figure 6c, the pinhole point cloud is generated by interpreting the estimated depth values as *z* in the pinhole coordinate system. As shown in the figure, the lane markings on both sides of the road appear curved due to the influence of the cylindrical projection. In contrast, Figure 6d shows the cylinder point cloud, which interprets the estimated depth values as *r*, restoring the lane lines as straight and producing a structure that more closely reflects the actual road geometry.

**Figure 7:**
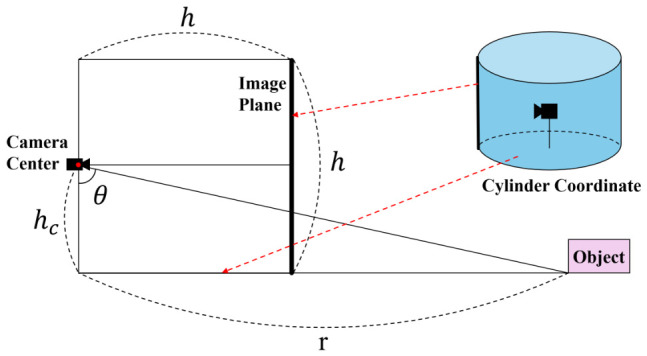
Radial distance *r* in the proposed camera geometry. The red arrows represent the height and the bottom of the cylinder coordinate system.

However, since the generated point cloud is constructed based on depth values estimated by a monocular depth model, it may still lack accurate scale information. To address this, we apply a correction step using camera geometry. Given that the front camera is installed such that its *x*–*z* plane is parallel to the ground plane, the feature points located on the road surface within its image can be used to obtain accurate *r* values, as shown in Equation (3) and Figure 7, provided that the camera height is known.

(3) $$\theta =\arctan\left(\frac{h}{v-\frac{h}{2}}\right),\;r=h_{c}\tan\left(\theta \right)$$

Equation (3) defines *v* as the *y*-coordinate of a feature point in the image space and *h* as the image height in pixels, while $h_{c}$ denotes the scale factor corresponding to the camera height, which is set to 1 m in all experiments. Using this method, we extract ground feature points from the lower half of the expanded bounding boxes in the front camera image and compute their corresponding *r* values. We then perform linear regression, treating the monocularly estimated depth values as the independent variable and the computed *r* values as the dependent variable. The resulting regression coefficient is then applied uniformly to all vehicle feature points and ground feature points in the front, right, and left camera images to compensate for scale. Although the depth values of vehicle feature points may be less accurate than those of ground points, they are included to mitigate pose ambiguity that can occur when aligning only planar ground points in 3D space. Since the regression coefficient is derived from the front camera, its application to the point clouds of the right and left cameras may introduce slight inaccuracies in depth. Therefore, an additional scale adjustment is performed during the point cloud alignment step to improve the final calibration accuracy.

### 3.5. Point Cloud Matching and Alignment for Extrinsic Parameter Estimation

We estimate the extrinsic parameters between cameras by aligning the 3D point clouds generated for each camera. Three-dimensional point clouds are generated based on the matched feature points obtained between the front and right, and the front and left cameras. Among them, the point cloud from the front camera is scale-corrected, while those from the right and left cameras are approximately scaled using the regression coefficient derived from the front camera’s ground points. As a result, the point clouds are roughly aligned in scale, allowing for real-scale estimation of extrinsic parameters through point cloud registration.

To align two point clouds, we use a method based on Singular Value Decomposition (SVD) [26]. Each point cloud is first translated such that its centroid is aligned with the origin. The optimal rotation matrix R is then estimated by minimizing the sum of squared distances between corresponding points, and the translation vector t is computed from the displacement between the original centroids. The resulting rotation R and translation t between the two point clouds are obtained as shown in (4).

(4a) $$\begin{array}{c} P \end{array}=\left[\begin{array}{c} p_{1}\\ p_{2}\\ \vdots \\ p_{N} \end{array}\right]\in R^{N\times 3},\;Q=\left[\begin{array}{c} q_{1}\\ q_{2}\\ \vdots \\ q_{N} \end{array}\right]\in R^{N\times 3}$$

(4b) $$\begin{array}{cc} P_{c} & =P-\bar{p},\;Q_{c}=Q-\bar{q} \end{array}$$

(4c) $$\begin{array}{cc} H & =P_{c}^{⊤}Q_{c},\;\left(U,\Sigma ,V^{⊤}\right)=\mathrm{SVD}\left(H\right) \end{array}$$

(4d) $$\begin{array}{cc} R & =VU^{⊤} \end{array}$$

(4e) $$\begin{array}{cc} t & =\bar{q}-R\bar{p} \end{array}$$

Here, P and Q denote the source and target point clouds, respectively, and $\bar{p}$ and $\bar{q}$ represent the centroids of P and Q. The source point cloud refers to the 3D point cloud generated from the right or left camera, while the target point cloud is the one generated from the front camera. $P_{c}$ and $Q_{c}$ are the centered 3D coordinate matrices of P and Q, obtained by translating their centroids to the origin.

However, the accuracy of this alignment is limited due to scale ambiguity in the point clouds generated from the right and left cameras. To address this, we employ two alternative methods to correct the scale before performing registration. The first method is similar to the previous approach, but instead of using a fixed scale factor, we normalize the source point cloud by adjusting its scale based on the relative distribution of points around its centroid. Specifically, as shown in (5), we compute the ratio of the mean distances from each point to its centroid in the source and target point clouds. This ratio is then used to scale the source point cloud prior to alignment. This is a simple heuristic adjustment, not the least-squares optimal solution [27], but it is computationally efficient and sufficient for our framework.

(5a) $$\begin{array}{cc} s & =\frac{\sum_{i=1}^{N}∥q_{i}-\bar{q}∥}{\sum_{i=1}^{N}∥p_{i}-\bar{p}∥} \end{array}$$

(5b) $$\begin{array}{cc} P_{s} & =sP \end{array}$$

After computing the scale factor, we scale the source point cloud P by *s*, and then replace P in (4) with the scaled point cloud $P_{s}$ for subsequent alignment. Since the point cloud from the front camera is already scale-corrected, this process improves the scale accuracy of the point clouds from the right and left cameras by aligning them to the scale of the front camera’s point cloud.

The second method performs a nonlinear least-squares optimization [28] to jointly refine the extrinsic parameters, the global scale factor, and the per-point radius values $r_{i}$. This optimization uses the rotation matrix and translation vector obtained from the initial SVD-based alignment (4) as the initial transformation. Let *s*, r, and t denote the scale factor, rotation vector, and translation vector, respectively, and let R(r) be the rotation matrix parameterized using the Rodrigues formula. Each radius value $r_{i}$ represents the circular distance from the camera axis and is used to reconstruct the 3D point $p_{i}\left(r_{i}\right)$ in the cylindrical coordinate system. The objective is to find the optimal parameters that minimize the total cost function defined as a weighted combination of two losses: a geometric alignment loss and a planar consistency loss. The geometric alignment loss, $L_{\mathrm{geom}}$ in (6), minimizes the point-to-point distances between the transformed source points and their corresponding target points.

(6) $$L_{\mathrm{geom}}=\sum_{i=1}^{N}\rho \;\left({∥\;s\;R\left(r\right)\;p_{i}\left(r_{i}\right)+t-q_{i}∥}_{2}\right)$$

To further enforce structural consistency with the ground plane observed from the front camera, we introduce a planar constraint term $L_{\mathrm{plane}}$ in (7). The reference plane is defined by the normal vector n and offset *d*, which are obtained by fitting a plane to the 3D points corresponding to the ground feature points detected in the front camera image. This term penalizes deviations of the transformed points from the fitted plane.

(7) $$L_{\mathrm{plane}}=\sum_{j=1}^{M}\rho \;\left(n^{⊤}\;\left(s\;R\left(r\right)\;p_{j}\left(r_{j}\right)+t\right)+d\right)$$

The two loss terms are normalized by their respective numbers of residuals, 3N for the geometric term and *M* for the planar term, to balance their relative magnitudes, as combined in (8), since each geometric residual consists of three components along the *x*, *y*, and *z* axes, whereas each planar residual represents a single scalar distance from the ground plane. This normalization ensures that both loss terms contribute comparably to the optimization, preventing either term from dominating the total objective. The overall cost function is minimized using the least_squares solver from the SciPy optimization library [29], which performs nonlinear least-squares fitting with robust loss functions.

(8) $$L_{\mathrm{total}}=\alpha \;L_{\mathrm{geom}}+\beta \;L_{\mathrm{plane}},\;\alpha =\frac{1}{\sqrt{3N}},\;\;\beta =\frac{1}{\sqrt{M}}$$

In these loss terms, we employ the Huber loss ρ(·) [30] to reduce the influence of outliers, which adaptively transitions between $l_{2}$ and $l_{1}$ norms depending on the magnitude of the residuals, as defined in (9).

(9) $$\rho \left(x\right)=\left\{\begin{array}{cc} \frac{1}{2}x^{2} & \mathrm{if}\;|x|\leq \delta \\ \delta \left(\left|x\right|-\frac{1}{2}\delta \right) & \mathrm{if}\;|x|>\delta \end{array}\right.$$

δ is the threshold parameter for the loss transition, and we set δ=1.0. Furthermore, we constrain the optimization ranges of $r_{i}$ and *s* to within ±50% of their initial values to prevent convergence to meaningless solutions with low residuals but excessively varying parameters.

## 4. Experiments

### 4.1. Qualitative Experiment with Real Data

To evaluate the performance of the proposed method, we have done experiments using both real-world and synthetic data. This section first describes the experiments using the real-world data. Since obtaining accurate GT camera poses in real-world environments is difficult, we conducted a qualitative evaluation based on visual inspection. As mentioned in an earlier section, the experiment was conducted using three cameras mounted on a vehicle, and driving videos were recorded at 15 fps while circling a parking lot on a university campus. In this real-data setup, the camera streams were synchronized using a 10 ms tolerance, and frames were paired by timestamp (maximum inter-camera offset of 10 ms). We used the same cameras, DFK 33UP1300 from Imaging Source (Charlotte, NC, USA), equipped with HFE1414C fisheye lenses from SENKO (Tokyo, Japan). Each camera’s resolution was set to 1280 × 1024 pixels, and the lenses provided a wide field of view of 185°. The front camera was mounted at a height of 1 m, with its *x*–*z* plane aligned parallel to the ground, while the left and right cameras were rotated approximately 60° outward from the front camera without any additional pose constraints. The EUCM parameters for all three cameras were calibrated using the Kalibr toolbox.

**Figure 8:**
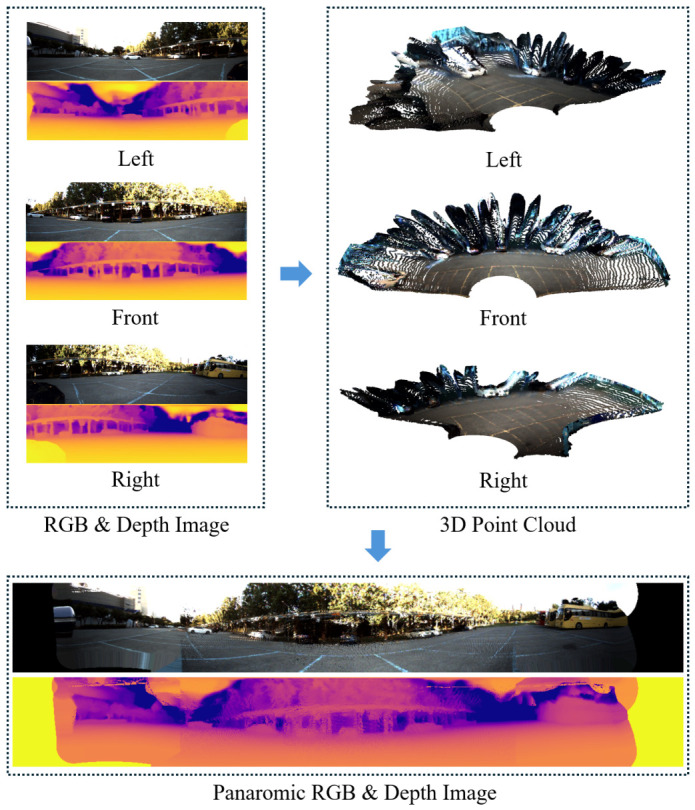
Generation of panoramic RGB and Depth image from three cameras.

We applied the proposed method to the driving video to estimate the extrinsic parameters between the front and right cameras, and between the front and left cameras. To evaluate the accuracy of the estimated camera poses, we made a warped cylinder image. First, depth was estimated using the SPIdepth model from the initial frame of each of the three cameras, after which all pixels of the cylindrical images were projected into 3D space. Using the estimated extrinsic parameters, we transformed the point clouds from the right and left cameras into the coordinate system of the front camera. These transformed point clouds were then projected onto a 360° cylindrical image plane aligned with the front camera’s coordinate system to generate the final RGB and depth warping results, as shown in Figure 8. The alignment results demonstrate structural consistency in the overlapping regions of the three cameras, visually validating the accuracy of the estimated extrinsic parameters.

The real-world experiment demonstrates structurally consistent alignment on actual driving videos, but the evaluation remains qualitative because reference extrinsic parameters were not available for the field sequence. A quantitative real-world evaluation would require independent reference calibration using artificial calibration targets under the same fixed camera mounting configuration. This field-to-lab validation protocol remains an important direction for further validation.

### 4.2. Construction of Synthetic Dataset

**Figure 9:**
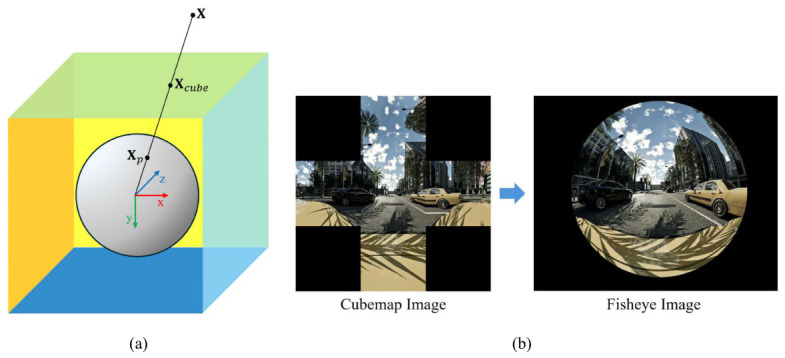
(**a**) 3D relationship between cubemap and fisheye; (**b**) Conversion from cubmap image to fisheye image.

Because accurate ground-truth extrinsic parameters are difficult to obtain for real driving sequences, and because no open benchmark dataset is available for fisheye-camera extrinsic calibration, we used a synthetic dataset for quantitative evaluation. To create our custom dataset, we used the CARLA simulator (version 0.10.0), which provides significantly enhanced visual realism compared to earlier CARLA releases, including the version used for the SynWoodScape dataset [31]. To generate fisheye images in CARLA, we followed the design strategy of SynWoodScape. First, a virtual fisheye camera center was defined, and five undistorted pinhole cameras sharing the same center were placed in the upward, downward, leftward, rightward, and forward directions to capture images. These five images were combined into a cubemap, which was then mapped into a fisheye image. The overall projection relationship is illustrated in Figure 9a. In Figure 9a, X is a world point in the camera coordinate system, $X_{cube}$ is the point coordinates when the five pinhole images are arranged as a 3D cubemap, and $X_{p}$ represents the point on the fisheye projection surface. Using the EUCM projection model, pixel points on the cubemap image can be mapped to corresponding points on the fisheye image, thereby producing a synthetic fisheye image. This process is equivalent to replacing the cylindrical plane in Figure 3 with a cubemap and applying a 3D-to-2D projection.

Examples of the cubemap and the resulting fisheye image are shown in Figure 9b. The intrinsic parameters used for the EUCM projection were the same as those used for the three real cameras in the previous section. The resulting synthetic fisheye cameras were mounted on the ego vehicle at the front, right, and left positions. The front camera was placed at the ego vehicle coordinate (2.5, 0, 1), facing forward. The right camera was placed at (1, 1, 1) and rotated by +60° about the vehicle’s *z*-axis, and the left camera was positioned at (1, −1, 1) and rotated by −60° about the same axis. And all cameras are installed with the camera’s *x*–*z* plane parallel to the ground. And we also attached 3D LiDAR at the ego vehicle coordinate (0, 0, 2). Subsequently, we used the Town10HD map in CARLA, where we spawned 40 autonomous vehicles and activated autopilot mode. The ego vehicle also operated in autopilot mode, driving through the map while collecting data. Each camera captured 1000 images at a frame rate of 1 fps during driving. The LiDAR also collected 1000 synchronized scans during the image acquisition process.

### 4.3. Comparison of Depth Interpretation

We applied a pinhole-based monocular depth model, SPIdepth, to the cylindrical images and performed post-processing to estimate the depth values and reconstruct 3D point clouds. To evaluate the accuracy of the monocular depth model and the reconstructed geometry, we conducted experiments using data generated from the CARLA simulator. Depth values were first estimated from the cylindrical images of the three cameras using SPIdepth. To avoid unreliable depth estimates, the regions containing the ego vehicle body and the upper half of the sky region above the horizon were excluded from the evaluation. LiDAR data were then projected onto the corresponding camera coordinates, and the projected depths were compared with the monocular depth estimates for quantitative evaluation. The LiDAR points were interpreted in three different ways: (1) the conventional z-axis depth of the camera, (2) the proposed r value defined in our method, (3) the refined r value obtained using ground-depth refinement. The quantitative results are summarized in Table 1. The table presents the evaluation metrics: AbsRel, RMSE, RMSE_log_, and the inlier ratio. The results were obtained using both median scaling and RANSAC-based [32] scale alignment, where τ denotes the error bound of RANSAC.

As shown in Table 1, interpreting monocular depth as *r* yields consistently better performance than using the conventional *z* value. Specifically, in the RANSAC-based evaluation, the inlier ratio is substantially higher for *r* than for *z*. In the cylindrical depth images, the central region resembles the pinhole projection, whereas the side regions exhibit significant geometric discrepancies. Due to these geometric distortions, the *z*-based interpretation produces similar overall error values but shows a markedly lower inlier ratio in the side regions. The non-RANSAC evaluation also indicates that the *r*-based interpretation performs better in terms of AbsRel and RMSE_log_, suggesting that *r* more accurately represents the underlying 3D scene structure. Furthermore, the refined *r* value improves the inlier ratio even further, reaching nearly all valid projected LiDAR points in several settings. This indicates that the ground-depth refinement makes the estimated depth more geometrically consistent with the visible 3D scene, even when RMSE-based metrics are less sensitive to this improvement due to remaining projection mismatches and outliers.

### 4.4. Quantitative Evaluation Using Synthetic Dataset

We evaluated the accuracy of the proposed method using the known ground-truth camera poses associated with the collected dataset. Since the dataset was generated in CARLA, the front/right/left images at each frame were captured synchronously at the same simulator tick, providing ideal time synchronization for quantitative evaluation. For each camera, we extracted 17 sliding windows of 200 consecutive frames by setting the starting frame indices to 0, 50, 100, 150, …, from the 1000 collected frames. Using these windows, we evaluated the proposed method with 200 images per camera (600 images in total across three cameras), repeatedly estimating the extrinsic parameters between camera pairs. For comparison, we also evaluated two widely used camera pose estimation frameworks: COLMAP and DUSt3R. Since COLMAP does not support the EUCM model, we re-estimated the intrinsic parameters from the same calibration dataset using the OpenCV fisheye model in Kalibr and applied these parameters to COLMAP. The same set of 600 images was used as input to COLMAP, and the relative poses estimated for each frame were averaged to obtain the final evaluation results. As DUSt3R is trained on pinhole images, we did not evaluate it directly on fisheye images. Instead, we projected the fisheye images into the pinhole coordinate system with the same resolution.

To ensure sufficient overlap between the front, right, and left cameras, we generated wide-angle pinhole images with a field of view of approximately 143.13°, instead of using the 90° FOV of a standard cubemap face, which lacks overlap between views. Although we initially attempted to input all 600 images into DUSt3R, memory limitations prevented successful execution. Therefore, we used triplets of front, right, and left images as input for each frame, estimated the extrinsic parameters for each triplet, and computed the average pose error over all 200 triplets for evaluation.

We evaluated three versions of our proposed method: (1) the basic SVD-based algorithm without scale correction, (2) with scale correction based on the relative distance between point cloud centroids and points, (3) using joint optimization of global scale and depth value. The quantitative results are summarized in Table 2. To examine the influence of the camera projection model on the proposed method, we also evaluated the calibration performance using the Double Sphere (DS) model [4] instead of EUCM. The corresponding results are presented in Table 3.

The evaluation was conducted using two metrics: Rotation Error and Translation Direction Error. Rotation Error measures the angular difference between the estimated and ground-truth rotation matrices. Translation Direction Error evaluates the angular difference between the estimated and ground-truth translation vectors, considering only their directions. This directional measure is required because COLMAP and DUSt3R do not recover the absolute translation scale.

Among all methods, the optimization-based version of the proposed algorithm achieved the best performance. DUSt3R exhibited the lowest performance, mainly due to the limited field of view of pinhole images and the severe distortions resulting from widening the view to achieve sufficient overlap. While COLMAP performed better than DUSt3R due to its native support for fisheye images, its overall performance remained limited. This is mainly because COLMAP relies on SfM, which requires densely overlapping image sequences. In contrast, our dataset was collected at 1 fps with wide-baseline camera pairs, resulting in insufficient overlap for reliable SfM-based estimation. Additionally, road-driving scenes often contain large low-texture regions, which further degrade feature matching and adversely affect COLMAP’s performance.

In contrast, the proposed method performed robustly even under these conditions. By using cylindrical images that preserve the wide field of view of fisheye cameras, the method maintained sufficient overlap even in wide-baseline settings. It also remained stable under low frame rates by accumulating feature matches across frames, rather than relying on dense temporal continuity between consecutive frames. The basic version of our method, which directly used 3D point clouds without scale correction, showed limited performance due to scale inconsistencies in the left and right camera point clouds. The optimization-based version addressed this issue by jointly refining the global scale and the individual *r* values of matched points, leading to a substantial improvement in calibration accuracy. In particular, the rotation error was reduced to below approximately 1°, indicating accurate rotation estimation. However, the translation direction error remained relatively larger, likely because the method relies on monocular depth estimation. Since monocular depth can contain scale and local geometric inaccuracies, the reconstructed 3D point positions may be distorted, making accurate translation direction estimation more challenging.

Building on the results obtained using the EUCM model, we further examined whether the performance remains stable under a different fisheye camera model. As shown in Table 3, the performance obtained using the DS model is highly consistent with that achieved using the EUCM model. This confirms that the proposed method does not rely on a specific fisheye projection model and remains robust as long as the projection geometry is properly handled during cylindrical projection and 3D reconstruction. Therefore, the proposed pipeline can be considered applicable to various fisheye camera configurations in practical scenarios.

### 4.5. Sensitivity Analysis

**Figure 10:**
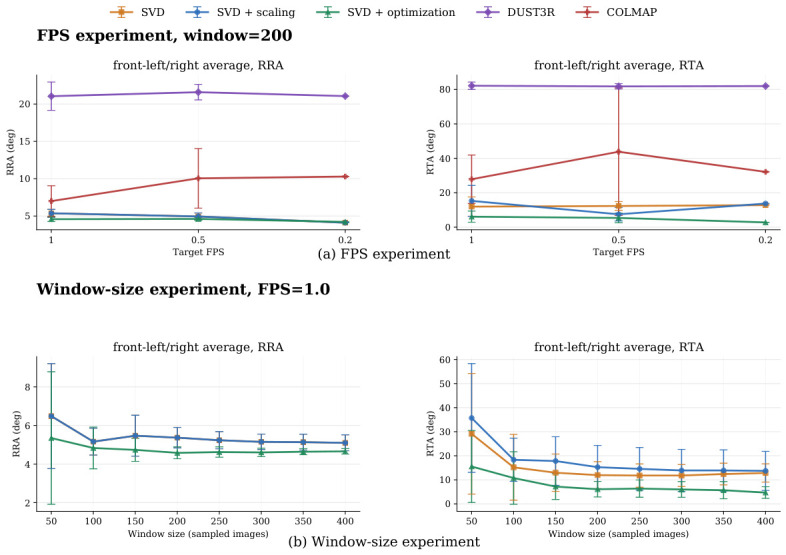
This figure shows the result of the sensitivity analysis of frame rate and data quantity.

We conducted sensitivity analyses to evaluate the robustness of the algorithms to changes in temporal sampling rate and estimation window size. The results are shown in Figure 10. In the FPS and window-size analyses, the errors from the front-left and front-right camera pairs were averaged before reporting.

In the FPS sensitivity test, DUST3R showed only minor changes across different sampling rates, since it estimates the relative camera configuration from the front, left, and right images captured at each timestamp rather than relying on temporal correspondences. COLMAP achieved comparable mean accuracy in some settings, but its standard deviation increased as the frame interval became larger, indicating higher sensitivity to sparse temporal sampling. The result at 0.2 FPS should be interpreted with caution because only one complete 200-frame evaluation window was available, which can artificially reduce the reported standard deviation. In contrast, the proposed methods maintained nearly constant mean error and standard deviation across FPS settings, suggesting stable performance under different temporal sampling rates when sufficient observations are available.

We also evaluated the effect of the estimation window size for the proposed methods. A 50-frame window resulted in larger error and variance, indicating that the number of feature correspondences was insufficient for stable calibration. Increasing the window size reduced the error, but the improvement became marginal after approximately 200 frames. Therefore, a 200-frame window was used as the default setting in the main experiments, balancing accuracy, stability, and computational cost.

### 4.6. Evaluation Under Additional Camera Rig Configurations

We further evaluated the optimization method under different camera rig settings to examine its applicability beyond the original camera configuration. The front camera was fixed as the reference view, while the side-camera height, longitudinal position, and yaw angle were changed from the original configuration. The original setting corresponds to x=1.0, z=1.0, and side-camera yaw of 60°. Here, the *x*-axis denotes the forward direction of the vehicle, and the *z*-axis denotes the upward vertical direction. The tested settings were selected to represent additional camera placement scenarios rather than applying the same displacement magnitude to each parameter. The tested settings were selected as representative camera rig changes rather than identical perturbations for all parameters. The side-camera height was varied over a larger range because it is directly related to the ground-based depth scale correction, while the longitudinal position was varied within a smaller local range around the original configuration.

As shown in Table 4, the proposed method remained stable under several different camera rig settings. In particular, the x=1.1 and yaw 50° settings produced errors comparable to, or slightly better than, the original configuration. This is likely because these settings increase the image overlap between the front and side cameras: the x=1.1 setting places the side cameras closer to the front camera, and the yaw 50° setting makes the side cameras face more toward the front view. The increased overlap can provide more reliable correspondences, resulting in stable or improved calibration accuracy.

The main degradation was observed when the side-camera height was changed. In both the z=0.8 and z=1.2 settings, the translation direction error increased for both the front-right and front-left camera pairs, while the rotation error remained relatively low. This result is closely related to the depth correction strategy used in the proposed method. The depth scale correction is computed based on the front camera, assuming that its height is known and that the camera is parallel to the ground. Under this assumption, the depth of ground points can be estimated and used to correct the monocular depth scale. However, when the heights of the side cameras are changed, applying the same correction factor to the side-camera point clouds can introduce scale errors because their ground geometry no longer follows the original height assumption. These scale errors directly affect the reconstructed 3D point positions, making translation direction estimation more difficult.

The results also show that cases with large differences between the front-right and front-left pairs tended to have worse performance in the front-right pair. For example, the x=0.9 setting produced a larger rotation error in the front-right pair, while the yaw 70° setting produced a much larger translation direction error in the front-right pair than in the front-left pair. This tendency can be explained by the correspondence statistics in Table 5. The front-right pair had slightly fewer matched feature points and smaller object bounding boxes than the front-left pair, indicating that the front-right pair provides weaker visual and geometric constraints for calibration. Therefore, when the camera placement reduces image overlap or changes the relative viewing geometry, the front-right pair is more likely to suffer from unstable rotation or translation direction estimation.

These results show that the proposed method can be applied to different camera rig settings, although its accuracy is affected by the validity of the depth scale correction and the quality of visual correspondences available in each camera pair.

### 4.7. Runtime Evaluation

We evaluated the runtime on a desktop equipped with a single NVIDIA RTX 4500 Ada Generation GPU. The reported runtime corresponds to the time required to evaluate a single calibration result. The evaluation was performed over all 17 sliding image windows, and we report the mean and standard deviation of the measured runtime as shown in Table 6. The average runtime remains under three minutes, indicating the practical applicability of the proposed method.

### 4.8. Practical Deployment Conditions and Limitations

Although the proposed method does not require artificial calibration targets, it still depends on several practical conditions. Accurate intrinsic calibration is required because cylindrical projection and 3D point reconstruction directly rely on the camera model parameters. Sufficient overlap between the front and side cameras is also necessary, since the method estimates extrinsic parameters from matched vehicle instances and local feature correspondences in the overlapping regions. Small overlap, large relative yaw angles, or weak texture can reduce reliable correspondences and make point-cloud alignment unstable, particularly for translation estimation.

Inter-camera synchronization is another important factor. Larger timestamp offsets may cause moving vehicles to appear at different positions across views, degrading both object-level and feature-level matching. The method also depends on the reliability of monocular depth estimation in cylindrical image space. Domain shift, reflective surfaces, occlusions, poor lighting, non-planar roads, inaccurate ground assumptions, or camera height differences can introduce scale errors. Future improvements include longer observation windows, stricter geometric verification, depth-uncertainty weighting, improved synchronization, and camera-specific or height-aware depth correction.

## 5. Conclusions

This paper presented a novel targetless calibration method for estimating the extrinsic parameters between multiple fisheye cameras mounted around a vehicle using driving videos. By projecting fisheye images into a cylindrical coordinate system, pre-trained pinhole-based models—YOLOv8 for object detection and SPIdepth for monocular depth estimation—are directly applied without additional retraining. Feature correspondences were established by matching detected vehicle instances across views and converted into 3D point clouds using the estimated depth values. These depth values were subsequently corrected based on the front camera, whose *x*–*z* plane was assumed to be parallel to the ground. A joint optimization of depth and scale was then performed to align the reconstructed 3D point clouds and estimate the inter-camera extrinsics.

The proposed method enables extrinsic calibration without artificial targets and demonstrates robustness under wide-baseline configurations and low-frame-rate conditions. Experimental results confirmed that fisheye images, once transformed into cylindrical projections, can be effectively processed by models trained on conventional pinhole imagery. Moreover, the optimization-based method achieved accurate rotation estimation, with rotation errors reduced to approximately 1° or lower. However, translation direction estimation remained more challenging due to scale and local geometric inaccuracies in monocular depth estimation.

Future work will focus on improving the proposed method under more diverse real-world deployment conditions. First, feature extraction and depth estimation can be improved by adapting detection and depth models to cylindrical images. Second, the depth correction strategy can be extended to account for side-camera height and non-planar road geometry. Since the current method applies a single correction factor estimated from the front camera to the side-camera point clouds, height differences can introduce scale errors. Future work could reduce this issue by estimating a height-aware depth correction for each side camera based on the consistency of reconstructed point clouds across camera pairs. Finally, the framework will be extended to rear-facing cameras and jointly optimize all camera pairs toward a fully automated 360° surround-view calibration system.

## Figures and Tables

**Table 1:** Depth evaluation under three depth value interpretations (*z*, *r*, and refined *r*). *z* denotes the camera-axis depth, *r* denotes the radial distance, and refined *r* denotes the radial depth after ground-depth refinement.

Method	Type	Camera	AbsRel ↓	RMSE ↓ (m)	RMSE_log_ ↓	Inlier ↑
Median Scaling	*z*	Front	0.450	5.146	0.520	-
		Right	0.561	4.359	0.625	-
		Left	0.572	4.590	0.623	-
	*r*	Front	0.237	4.977	0.330	-
		Right	0.428	4.731	0.515	-
		Left	0.417	4.882	0.500	-
	$r_{\mathrm{refined}}$	Front	0.237	6.198	0.425	-
		Right	0.402	5.065	0.537	-
		Left	0.398	5.295	0.523	-
RANSAC (±0.20)	*z*	Front	0.520	4.755	0.536	0.351
		Right	0.664	4.100	0.640	0.350
		Left	0.707	4.224	0.647	0.351
	*r*	Front	0.219	5.601	0.366	0.555
		Right	0.415	4.781	0.520	0.553
		Left	0.411	4.953	0.503	0.559
	$r_{\mathrm{refined}}$	Front	0.232	6.100	0.414	0.988
		Right	0.400	5.055	0.538	0.948
		Left	0.398	5.247	0.520	0.977
RANSAC (±0.30)	*z*	Front	0.547	4.670	0.546	0.481
		Right	0.694	4.076	0.650	0.467
		Left	0.736	4.203	0.656	0.465
	*r*	Front	0.235	5.011	0.333	0.753
		Right	0.438	4.631	0.516	0.705
		Left	0.442	4.718	0.500	0.719
	$r_{\mathrm{refined}}$	Front	0.232	6.095	0.414	0.999
		Right	0.400	5.043	0.536	0.975
		Left	0.398	5.241	0.519	0.992

**Table 2:** Rotation and translation direction error between camera pairs (EUCM).

Method	Rotation Error (deg)		Translation Direction Error (deg)	
	Front-Right	Front-Left	Front-Right	Front-Left
DUSt3R [23]	21.547 ± 1.961	20.557 ± 1.876	86.833 ± 11.957	77.538 ± 11.013
COLMAP [24]	7.268 ± 1.427	6.430 ± 3.363	27.962 ± 14.990	29.787 ± 21.911
SVD-based method	2.294 ± 1.452	1.689 ± 0.932	10.359 ± 5.377	12.664 ± 7.209
SVD-based method + scaling	2.294 ± 1.452	1.689 ± 0.932	22.480 ± 14.011	9.938 ± 7.560
SVD-based method + optimization	0.717 ± 1.272	0.832 ± 0.605	5.268 ± 4.558	6.039 ± 3.690

**Table 3:** Rotation and translation direction error between camera pairs (DS).

Method	Rotation Error (deg)		Translation Direction Error (deg)	
	Front-Right	Front-Left	Front-Right	Front-Left
SVD-based method	1.538 ± 1.021	1.891 ± 1.101	7.646 ± 5.820	13.405 ± 11.175
SVD-based method + scaling	1.538 ± 1.021	1.891 ± 1.101	36.097 ± 22.840	15.105 ± 8.977
SVD-based method + optimization	0.912 ± 1.014	0.643 ± 0.378	7.418 ± 5.354	5.400 ± 4.115

**Table 4:** Calibrationresults of the optimized refinement algorithm across different camera settings.

Setting	Rotation Error (deg)		Translation Direction Error (deg)	
	Front-Right	Front-Left	Front-Right	Front-Left
Original	0.717 ± 1.272	0.832 ± 0.605	5.268 ± 4.558	6.039 ± 3.690
z=0.8	1.621 ± 0.842	1.066 ± 0.614	15.941 ± 6.749	16.250 ± 4.349
z=1.2	0.419 ± 0.341	0.710 ± 0.589	15.460 ± 3.295	14.827 ± 2.559
x=0.9	1.260 ± 3.004	0.593 ± 0.391	8.279 ± 6.561	4.075 ± 2.344
x=1.1	0.420 ± 0.243	0.380 ± 0.221	6.226 ± 2.927	2.586 ± 1.445
Yaw 50°	0.654 ± 1.292	0.493 ± 0.362	5.337 ± 5.843	4.161 ± 2.601
Yaw 70°	0.897 ± 0.770	0.961 ± 0.684	12.919 ± 9.759	5.888 ± 2.834

**Table 5:** Calibration results of the optimized refinement algorithm across different camera rig settings. Original denotes the default setting with x=1.0, z=1.0, and side-camera yaw of 60°.

Metric	Front-Right	Front-Left
Number of matched feature points	50.41	53.41
Bounding-box width (pixel)	79.20	100.15
Bounding-box height (pixel)	36.00	38.79

**Table 6:** Runtime evaluation results (unit: s).

	EUCM	Double Sphere
Synthetic Data	170.055 ± 26.897	168.731 ± 27.316
Real Data	172.078 ± 6.019	172.100 ± 3.546

## Data Availability

The raw data supporting the conclusions of this article will be made available by the authors on request.
